# Imaging Findings in Patients With H1N1 Influenza A Infection

**DOI:** 10.5812/iranjradiol.4554

**Published:** 2011-12-25

**Authors:** Mehrdad Bakhshayeshkaram, Bahareh Saidi, Payam Tabarsi, Soheila Zahirifard, Mishka Ghofrani

**Affiliations:** 1 Department of Radiology, Pediatric Respiratory Disease Research Center, National Research Institute of Tuberculosis and Lung Diseases (NRITLD), Masih Daneshvari Hospital, Shahid Beheshti University of Medical Sciences, Tehran, Iran; 2 Department of Infectious Diseases, Clinical Tuberculosis and Epidemiology Research Center, National Research Institute of Tuberculosis and Lung Diseases (NRITLD), Masih Daneshvari Hospital, Shahid Beheshti University of Medical Sciences, Tehran, Iran; 3 Department of Infectious Diseases, Mycobacteriology Research Center, National Research Institute of Tuberculosis and Lung Diseases (NRITLD), Masih Daneshvari Hospital, Shahid Beheshti University of Medical Sciences, Tehran, Iran; 4 Department of Radiology, Erfan Hospital, Tehran, Iran; 5 Department of Radiology, Lung Transplantation Center, National Research Institute of Tuberculosis and Lung Diseases (NRITLD), Masih Daneshvari Hospital, Shahid Beheshti University of Medical Sciences, Tehran, Iran

**Keywords:** Radiography, Thoracic, Tomography, X-Ray Computed, Influenza A Virus, H1N1 Subtype, Infection

## Abstract

**Background:**

Swine influenza (H1N1) is a very contagious respiratory infection and World Health Organization (WHO) has raised the alert level to phase 6 (pandemic). The study of clinical and laboratory manifestations as well as radiologic imaging findings helps in its early diagnosis.

**Objectives:**

The aim of this study was to evaluate the imaging findings of patients with documented H1N1 infection referred to our center.

**Patients and Methods:**

Thirty-one patients (16 men) with documented H1N1 infection were included in our study. The initial radiography obtained from the patients was reviewed regarding pattern (consolidation, ground glass, nodules and reticulation), distribution (focal, multifocal, and diffuse) and the lung zones involved. Computed tomography (CT) scans were also reviewed for the same abnormalities. The patient files were studied for their possible underlying diseases.

**Results:**

The mean age was 37.97 ± 13.9 years. Seventeen (54.8%) patients had co-existing condition (eight respiratory, five cardiovascular, two immunodeficiency, two cancer, four others). Twelve (38.7%) patients required intensive care unit (ICU) admission. Five (16.1%) patients died. (25.8%) had normal initial radiographs. The most common abnormality was consolidation (12/31; 38.7%) in the peripheral region (11/31; 35.5%) followed by peribronchovascular areas (10/31; 32.3%) which was most commonly observed in the lower zone. The patients admitted to the ICU were more likely to have two or more lung zones involved (P = 0.005).

**Conclusions:**

In patients with the novel swine flu infection, the most common radiographic abnormality observed was consolidation in the lower lung zones. Patients admitted to ICU were more likely to have two or more lung zones involved.

## 1. Background

The new H1N1 influenza infection or the swine flu is a very contagious respiratory tract infection which came to attention in Mexico in April 2009 [1]. Since then it has rapidly spread in different countries and the World Health Organization (WHO) raised the alert level to phase 6 (pandemic level) by June 2009 [1]. Although most patients have mild symptoms and those with severe symptoms (sepsis, pneumonia, ARDS) mostly had an underlying diseases, it was recognized that H1N1 may affect young previously healthy individuals and unlike other subtypes of influenza may cause severe symptoms in this age group [2]. A case fatality rate (CFR) of 0.45% has been reported for this infection [3]. The patients may have different flu like manifestations such as fever, cough, sore throat, body aches, nausea and vomiting. The recognition of the pulmonary manifestations and radiologic features helps in early diagnosis, treatment and isolation of patients in order to prevent the spread of this very contagious respiratory tract infection.

Several studies have been conducted in order to obtain information regarding the chest X-ray and computed tomography (CT) scan findings and correlation of these manifestations with disease severity has been made. The most common radiologic findings were the opacities observed in the lower lung zones [1][4].

## 2. Objectives

In this study, we reviewed the radiologic manifestations in patients with documented H1N1 infection who were admitted to Masih Daneshvari Hospital to obtain the characteristic radiologic findings of H1N1 infection in Iran and to find any difference with previous studies if present.

## 3. Patients and Methods

In this retrospective study, the files of 54 patients admitted to Masih Daneshvari Hospital (a referral hospital for respiratory tract infection and diseases), from November 1 to December 30 2009, who had the criteria for H1N1 infection determined by US Centers of Disease Control and Prevention were reviewed. Most patients were referred from Northeast Tehran and the inclusion criteria were a body temperature above 37.8 ℃ (100 F), cough or sore throat and a real time reverse transcription-polymerase chain reaction (RT-PCR) positive for H1N1 infection. These patients were treated with oral antibiotics and oseltamivir. Thirty-one of the patients enrolled into the study had an available chest-X ray and ten patients with more severe symptoms had an available CT scan. The patients’ files were also reviewed regarding their previous medical condition and the laboratory findings including creatine phosphokinase (CPK), lactate dehydrogenase (LDH), hemoglobin (HB), leukocyte count and platelet count were recorded. Our institutional review board approved the study and the informed consent was waived as the study was retrospective and observational.

### 3.1. Radiologic Evaluation

Twenty eight patients had postero-anterior and lateral radiographs, of which three were anteroposterior portable bedside radiographs. The radiographs were obtained with a DDR/Axioma aristos VX plus, Siemens, Germany.

### 3.2. Image Analysis

One experienced radiologist in the field of thoracic imaging examined the radiographs. Whenever a previous radiograph was available, the new chest X-ray was compared to the previous one and if a new abnormality was present it was defined and recorded. The radiographs were studied regarding being normal or abnormal; unilateral or bilateral involvement and the pattern of involvement including ground glass, consolidation, reticulation and nodules. Ground glass opacities were considered when the background vascularity could be observed and consolidation when the underlying vascularity could not be observed. The presence of bilateral or unilateral lymph node enlargement in the mediastinum or hilum as well as bilateral or unilateral pleural thickening or effusion were assessed. The distribution of abnormalities was evaluated to be predominantly central, peribronchovascular or peripheral and focal, multifocal or diffuse. The distribution was also assessed in different lung zones by dividing into predominantly upper, middle or lower lung zone involvement. This was done in the frontal view with each lung divided into one thirds from the apex to the hemidiaphragm. The extent of lung zone involvement was compared in patients admitted to the intensive care unit (ICU) and patients in the ward. CT scans were also reviewed regarding the same abnormalities.

### 3.3. Statistical Analysis

Analysis was performed with SPSS version 15. The quantitative variables were determined by mean ± standard deviation (SD) and the categorical variables were shown with number and percentage. The extent of lung zone involvement comparison between the two groups was performed by chi-square. A P value < 0.05 was considered significant.

## 4. Results

**Figure 1:**
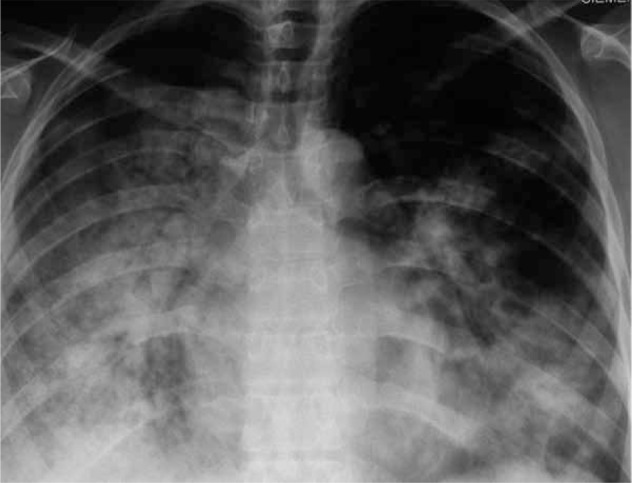
A 29-year-old female with respiratory symptoms and confirmed H1N1 infection. Chest x-ray shows extensive bilateral air-space opacities mainly in the lower zones.

**Figure 2:**
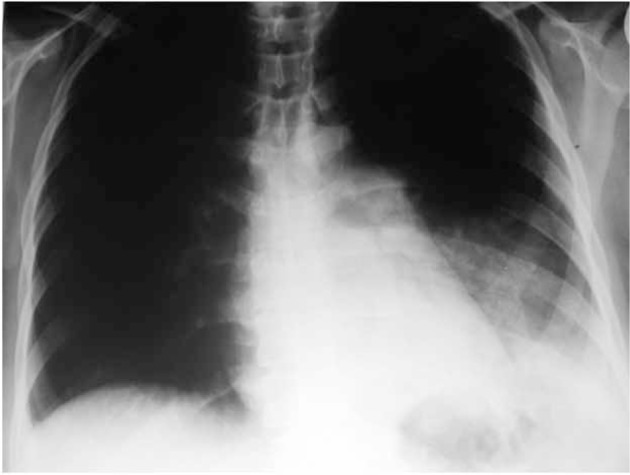
A 30-year-old female with respiratory symptoms and proved H1N1 infection. Chest x-ray demonstrates left pleural effusion. Pulmonary segment is also prominent.

**Figure 3:**
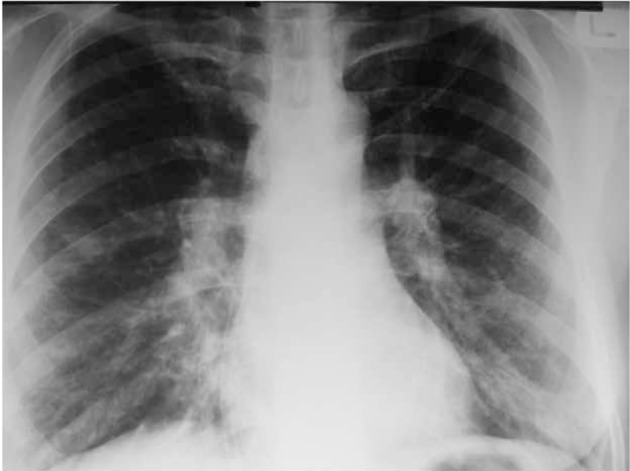
A 23-year-old female with respiratory symptoms and proved H1N1 infection, with crack addiction. Lymphadenopathy is shown on the chest x-ray.

The mean age was 37.97 ± 13.9 years (range, 18 to 76 years). There were 16 men (mean age, 30.06 ± 13) and 15 women (mean age, 40 ± 14.8 years). Seventeen (54%) had a co-existing medical condition (Table 1). The mean duration of stay in the hospital was 10.35 ± 9 days, range 2 to 50 days. Twelve (38.7%) patients were admitted to the ICU. Five (16.1%) died due to respiratory failure. Of our patients, 87.1% had fever, 58% cough, 80.6% dyspnea, 25.8% vomiting, 16.1% diarrhea and nine (29%) hemoptysis. Eight (25.8%) had normal radiographs. The abnormal pattern was unilateral in four (12.9%) and bilateral in 19 (61.3%). The most common abnormal radiographic pattern was consolidation (12/31, 38.7%) (Figure 1) observed most frequently in the peripheral region (11/31; 35.5%) followed by peribronchovascular infiltration (10/31; 32.3%) which was present in the left lower lung zone in 61.3% and the right lower lung zone in 45.2% (Table 2). Other findings included cardiomegaly in five (16.1%) patients. Five (16.1%) patients had bilateral and two (6.4%) had unilateral pleural thickening or effusion (Figure 2). Five (16.1%) had hilar or mediastinal adenopathy (Figure 3). CPK was elevated in 9/24 and LDH in 14/22 patient. The platelet count was low in 32.3%. Hb was low in 22.6%. White blood cell (WBC) was high in 22.6%. Twelve patients with a mean age of 39.5 (66.7% women) were admitted to ICU. Seven had a predisposing condition (three cardiovascular disease, two respiratory disease, one immunodeficiency, one cancer, three others). Four patients died. All had alterations on their radiographs, most commonly observed was multifocal (10/12; 83.3%) consolidation (6/12; 50%) in the lower zones (left 83.3%; right 66.7%). Four (33.3%) patients had more than two zones involved. Five (41.7%) had pleural thickening or effusion. Four (33.3%) had adenopathy. CPK was high in 6/10 and LDH in 10/10. The patients admitted to the ICU were more likely to have two or more lung zones involved (P = 0.005). Ten patients had an available CT scan and the most common pattern was ground glass opacities seen in six patients (60%) followed by consolidation in four (40%) which was most commonly observed in the peripheral region in six (60%) followed by the peribronchovascular region in five (50%) and in the upper lung zones in seven (70%) of patients.

**Table 1:** Patients Background Disease

**Co-existing Condition**	**No.**
Respiratory disease (Asthma, COPD^a^)	8
Cardiovascular (ischemic heart disease, hypertension)	5
Immunodeficiency (common variable immunodeficiency, HIV)	2
Cancer (multiple myeloma, synovial cell cancer)	2
Others (addiction, suicide, convulsions)	4

^a^ Abbreviation: COPD, chronic obstructive pulmonary disease

**Table 2:** Radiologic Findings of Patients With the New Swine Flu Influenza

	**NO. (%)**
Distribution	
Focal	8 (25.8)
Multifocal	16 (51.6)
Diffuse	1 (3.2)
Pattern	
Normal	8 (25.8)
Consolidation	12 (38.7)
Reticular	7 (22.6)
Ground glass	4 (12.9)
Nodules	1 (3.2)
Lung zones	
Left upper	9 (29)
Left middle	9 (29)
Left lower	19 (61.3)
Right upper	6 (19.4)
Right middle	4 (12.9)
Right lower	14 (45.2)
Pleural effusion	
Right	1 (3.2)
Left	1 (3.2)
Bilateral	5 (16.1)
Hilar or mediastinal lymphadenopathy	5 (16.1)

## 5. Discussion

By the end of April 2009, two cases of confirmed novel H1N1 were detected in the United States. These patients were resistant to rimantadine and amantadine and had no contact with swine. Further cases of the new swine flu were identified in Mexico and other countries. By June 2009, several confirmed cases were reported from 74 countries and the virus was known to have human-human transmission and by that time WHO raised the alert level to phase 6 which is the pandemic level [5]. The H1N1 influenza is a negative-sense RNA virus of the orthomyxoviridae family. The center for disease control and prevention (CDC) recognizes it with an influenza like syndrome presenting with high fever, cough or sore throat. Its diagnosis is confirmed by real-time reverse transcription polymerase chain reaction PCR or viral culture. Its incubation period is between 1 and 7 days. The patients are thought to be contagious from one day before to 7-10 days after the onset of the disease. Patients with a background disease including respiratory tract and heart disease are more likely to require hospitalization. The clinical presentations have been reported as fever, headache, sore throat, dyspnea, diarrhea and rhinorrhea. Laboratory findings are high CPK, high LDH and lymphopenia [1]. The new swine flu influenza S-OIV is known to be susceptible to neuraminidase inhibitors and there is recommendation to give oseltamivir as prophylaxis to the high risk group [2]. Different radiologic manifestations have been reported in several studies of the new swine flu influenza virus [4][6]. Perez Pallida [1] reported the radiologic manifestations of 18 patients with documented H1N1 infection as bilateral alveolar opacities which are predominantly basal and other observations being interstitial opacities (including linear and reticular). In a study on 66 patients, the most common abnormal pattern was consolidation most commonly observed in the lower and central lung zones and patients admitted to the ICU were more likely to have three or more lung zones involved [4]. This result was consistent with our study. The patients were more likely to have consolidations in the lower lung fields and those admitted to the ICU having two or more lung fields involved; however, in another study by Aviram et al. [7] performed on 97 patients who underwent chest radiography at admission, the most frequent abnormal pattern on radiography was ground glass opacities in the central and middle lung zones, which was followed by consolidation with slightly less frequency. This is in contrast with our findings which showed predominant involvement of the lower lung zones and consolidation as the most common manifestation. In their study, patients with bilateral and peripheral involvement or four or more lung zone involvement were more likely to have severe outcome, which is in consistence with our findings in patients admitted to ICU. It should be noted that our study population included patients with a more severe presentation and was not a sample of the population diagnosed with H1N1 and the results may only be interpreted in the setting where the manifestation is more severe and not the entire population of patients diagnosed with H1N1. Another study reviewed the High Resolution Computed Tomography Scan (HRCT) findings of 18 patients with the new swine flu influenza. In this study, the abnormal pattern was most commonly the ground glass opacity present in the peripheral region which is consistent with our results. In their study, patients with high LDH were more likely to have consolidations on HRCT [8].

Considering the association of chest findings and patients’ prognosis, it was previously demonstrated that in patients with community acquired pneumonia, bilateral pleural effusion may predict short term mortality [9]. In our group of patients, bilateral pleural effusion was not a predictor of mortality. Interestingly, in another report of patients with acute respiratory distress syndrome, involvement of more than two lung zones has been associated with the worst outcome [10]. In our group of patients, we also found that those who were admitted to the ICU were more likely to have more than two lung zones involved. Detection of multiple consolidations on radiography may represent a severe viral infection or superimposed bacterial infection which would necessitate antibiotic and some advocate administration of antibiotic to patients suspected of H1N1 and radiologic manifestation of extensive involvement or consolidation. In our group of patients, those with severe presentation were also receiving antibiotic alongside oseltamivir.

In conclusion, we found our experience with our group of patients with H1N1 influenza consistent with previous reports as consolidation on the lower lung fields being most common on radiography and ground glass opacities most common on the CT scan. Becoming familiar with the clinical and radiographic presentations of this very infectious disease helps in early diagnosis, treatment and isolation of patients.

## References

[1] Perez-Padilla R, de la Rosa-Zamboni D, Ponce de Leon S, Hernandez M, Quinones-Falconi F, Bautista E (2009). Pneumonia and respiratory failure from swine-origin influenza A (H1N1) in Mexico.. N Engl J Med.

[2] Dawood FS, Jain S, Finelli L, Shaw MW, Lindstrom S, Garten RJ (2009). Emergence of a novel swine-origin influenza A (H1N1) virus in humans.. N Engl J Med.

[3] (2009). World Health Organization.. Pandemic (H1N1)..

[4] Agarwal PP, Cinti S, Kazerooni EA (2009). Chest radiographic and CT findings in novel swine-origin influenza A (H1N1) virus (S-OIV) infection.. AJR Am J Roentgenol.

[5] Sullivan SJ, Jacobson RM, Dowdle WR, Poland GA (2010). 2009 H1N1 influenza.. Mayo Clin Proc.

[6] Cao B, Li XW, Mao Y, Wang J, Lu HZ, Chen YS (2009). Clinical features of the initial cases of 2009 pandemic influenza A (H1N1) virus infection in China.. N Engl J Med.

[7] Aviram G, Bar-Shai A, Sosna J, Rogowski O, Rosen G, Weinstein I (2010). H1N1 influenza: initial chest radiographic findings in helping predict patient outcome.. Radiology.

[8] Marchiori E, Zanetti G, Hochhegger B, Rodrigues RS, Fontes CA, Nobre LF (2010). High-resolution computed tomography findings from adult patients with Influenza A (H1N1) virus-associated pneumonia.. Eur J Radiol.

[9] Hasley PB, Albaum MN, Li YH, Fuhrman CR, Britton CA, Marrie TJ (1996). Do pulmonary radiographic findings at presentation predict mortality in patients with community-acquired pneumonia?. Arch Intern Med.

[10] Chau TN, Lee PO, Choi KW, Lee CM, Ma KF, Tsang TY (2004). Value of initial chest radiographs for predicting clinical outcomes in patients with severe acute respiratory syndrome.. Am J Med.

